# Diabetes

**Published:** 1871-10

**Authors:** 


### DIABETES.

The intolerable thirst attending this often fatal disease is said to be removed and the malady cured by drinking the water of the Bethesda Mineral Springs, where Col. Richard Dunbar is now erecting a hotel, the corner-stone of which was laid by Chief Justice Chase, August 24, 1871.

